# Virus Escape and Manipulation of Cellular Nonsense-Mediated mRNA Decay

**DOI:** 10.3390/v9010024

**Published:** 2017-01-23

**Authors:** Giuseppe Balistreri, Claudia Bognanni, Oliver Mühlemann

**Affiliations:** 1 Department of Biosciences, University of Helsinki, Helsinki FIN-00014, Finland; 2 Department of Chemistry and Biochemistry, University of Bern, Bern CH-3012, Switzerland; claudia.bognanni@dcb.unibe.ch; 3 Graduate School for Cellular and Biomedical Sciences, University of Bern, Bern CH-3012, Switzerland

**Keywords:** RNA quality control, gene expression, translation, RNA-protein interactions

## Abstract

Nonsense-mediated mRNA decay (NMD), a cellular RNA turnover pathway targeting RNAs with features resulting in aberrant translation termination, has recently been found to restrict the replication of positive-stranded RNA ((+)RNA) viruses. As for every other antiviral immune system, there is also evidence of viruses interfering with and modulating NMD to their own advantage. This review will discuss our current understanding of why and how NMD targets viral RNAs, and elaborate counter-defense strategies viruses utilize to escape NMD.

## 1. Introduction

### Virus Infections and Cellular mRNA Quality Controls

For their efficient replication, viruses rely on the host cell’s replicative machinery and must avoid being recognized by cellular antiviral responses. Having co-evolved with their hosts for millions of years, viruses have developed means to modulate cellular functions and redirect the metabolic resources of the infected cell to their advantage. Antiviral defense mechanisms, on the other hand, have arisen to counteract pathogen infections. Recent evidence indicates that cellular RNA quality control systems, such as the nonsense-mediated mRNA decay (NMD) pathway, can restrict viral infections by different mechanisms [1,2], suggesting that the cellular RNA decay machinery could act as an ancestral form of intrinsic antiviral immunity [3]. Supporting this hypothesis, there is increasing evidence that viruses have evolved mechanisms to interfere with or modulate NMD at different post‑entry stages of their replication cycle. In this review, we will analyze current evidence that supports a role of NMD in counteracting virus infections. To set the stage, we first describe the known features that make a cellular mRNA a substrate for NMD. Next, we apply this knowledge on viral RNAs and discuss how viral mRNAs could be recognized and attacked by the NMD surveillance machinery. Additionally, we will discuss which counter-defense strategies viruses utilize to ensure stability of their transcripts.

## 2. The Nonsense-Mediated mRNA Decay (NMD) Pathway

Eukaryotic cells possess numerous RNA quality control systems that degrade faulty mRNAs and so prevent the production of aberrant proteins. These mechanisms, including the NMD, the non-stop mRNA decay (NSD), and the no-go decay (NGD) pathways, are important for dynamically shaping the transcriptome and the proteome of eukaryotic cells to variable physiological conditions [1]. Of these mechanisms, NMD is arguably the best characterized [2]. NMD modulates the RNA levels of about 10% of all genes in diverse eukaryotes [3,4].

**Figure 1:**
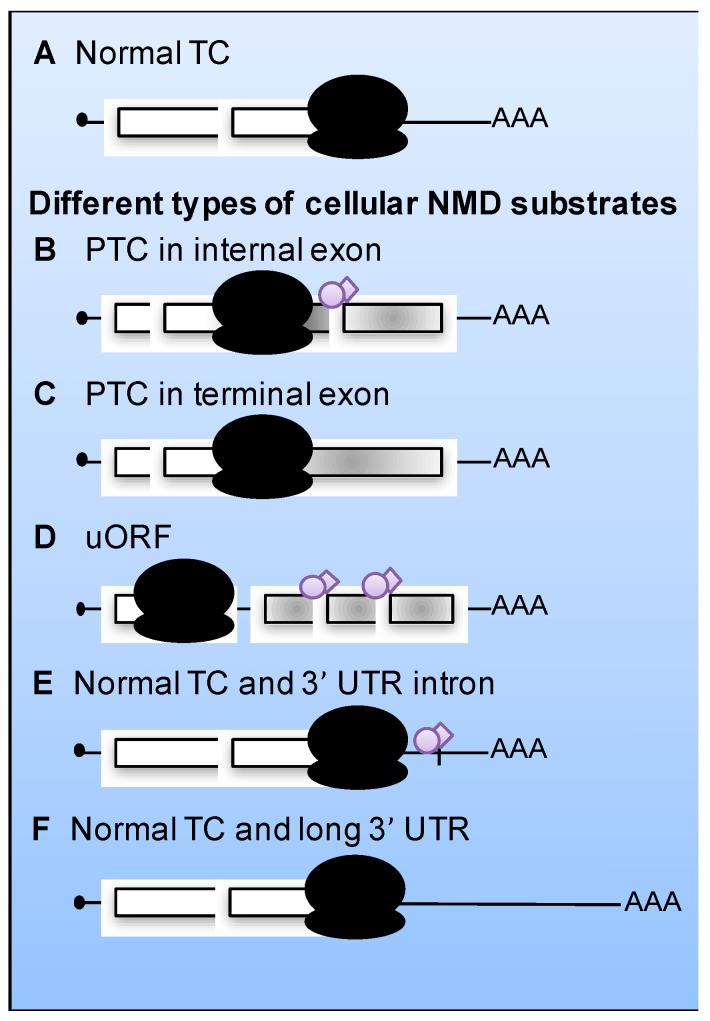
Different types of cellular mRNAs that can be substrates for nonsense-mediated mRNA decay (NMD). (**A**) An mRNA with a normal termination codon (TC) positioned in a context that does not trigger NMD: the termination codon is at the end of the last exon followed by a short 3’‑untranslated region (3’-UTR). (**B**–**F**) NMD targets comprise mRNAs with a truncated ORF due to a premature termination codon (PTC) in an internal (**B**) or terminal (**C**) exon. The presence of protein complexes known as exon junction complexes (EJCs) downstream the TC increases, but it is not necessary for, NMD (see Section 3). Upstream ORF (uORF), (**D**), the presence of EJC-associated introns in the 3’-UTR (**E**), and long 3’-UTR (**F**), all act as RNA destabilizing factors and trigger NMD. White boxes denote translated ORFs; gray boxes denote the fraction of the ORF that is not translated due to the presence of a PTC. Ribosomes are indicated in black, EJC in purple.

NMD was originally identified as an mRNA degradation pathway targeting transcripts that harbor a premature termination codon (PTC) [5,6,7]. However, in the last decade, it has become clear that NMD targets include a much broader number of substrates with different features. Transcripts targeted by NMD include those with PTCs in an internal exon or sometimes also in the terminal exon, as well as transcripts with long 3’-untranslated regions (UTRs), introns downstream a termination codon (TC) or upstream open reading frames (uORFs; Figure 1) [8,9,10,11]. The molecular details of how NMD is regulated and how NMD factors are recruited to target transcripts are not yet fully understood. It is clear that translation is a prerequisite for NMD and converging evidence suggests that a failure to terminate translation correctly triggers NMD [2]. This implies that any RNA molecule that engages with ribosomes to undergo translation is a potential target for NMD, including viral RNAs.

## 3. NMD Factors

As detailed in several recent reviews, the activation of NMD requires a set of evolutionarily conserved core regulatory factors called up-frameshift (UPF) 1 to 3 and additional factors in metazoans [2,11,12,13].

UPF1 is a monomeric superfamily 1 (SF1) helicase that is essential for substrate recognition and NMD execution. The helicase domain consists of two RecA domains that bind single-stranded RNA (ssRNA) and DNA (ssDNA). It is flanked by an N-terminal cysteine and histidine rich (CH) domain that binds UPF2 [14], the ribosomal protein RPS26 [15] and the decapping enzyme subunit DCP2 [16], and by an unstructured C-terminal domain enriched in serine-glutamine (SQ) dipeptides.

UPF2, the second conserved NMD factor, functions as a scaffold linking UPF1 and UPF3. Human UPF2 has three tandem MIF4G domains and a C-terminal UPF1 binding region. MIF4G-3 interacts with UPF3 while the specific functions of MIF4G-1 and MIF4G-2 are unknown [17]. Besides bridging UPF1 with UPF3, UPF2 functions as an activator of UPF1 by promoting its helicase activity, thereby switching UPF1 from the RNA clamping to the RNA unwinding mode [18]. 

Of the three UPF proteins, UPF3 is the least conserved one [7]. Two UPF3 versions encoded by two different genes exist in mammals, UPF3A and UPF3B. Both are predominantly nuclear and shuttle between the nucleus and the cytoplasm. They are associated with spliced mRNA and bind UPF2 and the exon junction complex (EJC) [19], a multi protein complex comprising four core components (eIF4A1, MAGOH, Y14 and MLN51) and more than a dozen other factors [20], which upon splicing assembles 24 nucleotides upstream of the spliced junction and remains associated with the mRNA until translation, thereby “marking” the exon-exon junction. 

Tethering experiments showed that UPF3A is less active than UPF3B in promoting NMD [21]. In human cells, UPF3B is the predominantly expressed protein and UPF3A becomes specifically upregulated if UPF3B levels are experimentally decreased or low because of a mutation [22]. Interestingly, a recent study shows that UPF3A is critical for spermatogenesis and provides evidence that it can antagonize NMD by sequestering UPF2, while its paralog, UPF3B activates NMD [23].

In addition to the three UPF factors, activation of NMD in metazoans requires the function of several additional proteins, among them a set of proteins originally identified in *Caenorhabditis elegans* known as the “suppressor with morphogenetic defects in genitalia” (SMG), SMG1 and SMG 5–9. These proteins control the phosphorylation and de-phosphorylation of UPF1 and trigger mRNA degradation by recruiting specific mRNA decay activities.

SMG1 belongs to the phosphatidylinositol 3 kinase-related protein kinase (PIKK) super family and it is responsible for phosphorylation of UPF1 at multiple S/TQ motifs [24]. SMG1, SMG8 and SMG9 form together with hypophosphorylated UPF1 and the eukaryotic release factors (eRF) 1 and 3 a complex called SURF [25,26], in which SMG8 and SMG9 repress the kinase activity of SMG1 [27]. Upon dissociation of SMG8 and SMG9 from SMG1, UPF2 mediates a structural rearrangement between SMG1 and UPF1 that activates the kinase, leading to UPF1 phosphorylation [28].

SMG5, SMG6 and SMG7 interact preferentially with hyperphosphorylated UPF1 and hence function further downstream in the NMD pathway. SMG5 and SMG7 form a heterodimer which interacts with phosphorylated SQ motifs in the C-terminal part of UPF1 and which recruits the CCR4‑NOT deadenylase complex onto the mRNA [29,30]. SMG6 by contrast appears to function as a monomer [31]. Its C-terminal PIN domain has endonuclease activity [32] and it interacts with UPF1 through phosphorylated T28 as well as phosphorylation-independent contacts in the helicase domain [33,34]. In addition to the abovementioned NMD factors, additional proteins have more recently been shown to be involved in NMD and their molecular function is currently being investigated [25,35,36,37].

## 4. Current NMD Model

### Aberrant Translation Termination Activates NMD

During normal translation termination, the ribosome when arriving at a termination codon (TC) binds eRF1 and eRF3. eRF1 recognizes the TC in the A-site of the stalling ribosome and forms a complex with the GTPase eRF3 to catalyze peptide release [38]. After eRF3-mediated GTP hydrolysis, eRF1 interacts with the ATP-binding cassette subunit family E member 1 (ABCE1). This interaction induces a structural change that stimulates ATP hydrolysis and leads to the separation of the two ribosomal subunits and the mRNA [39,40]. Importantly, in the context of NMD, the cytoplasmic polyA binding protein 1 (PABPC1) has recently been shown to enhance the recruitment of the eRFs, thereby stimulating correct and efficient translation termination [41]. Most likely due to this translation termination promoting activity, PABPC1 can potently antagonize NMD [42,43]. Based on these observations, it was postulated that whether an mRNA is subjected to NMD mainly depends on a competition between UPF1 and PABPC1 for binding to eRF3 [44]. The outcome of this competition is predicted to depend on the location of UPF1 and PABPC1 binding sites relative to the TC. Consistent with this model, TCs located in an mRNA towards the 3’ end in the vicinity of the polyA tail (i.e., PABPC1 binding sites) will usually not elicit NMD. In contrast, PTCs, which can occur anywhere in the mRNA, as well as long 3’ UTRs may allow UPF1 rather than PABPC1 to interact with eRF3 at the terminating ribosome and as a consequence activate NMD by formation of the SURF complex and subsequent UPF1 phosphorylation. Alternatively, if a TC is located more than 50 nucleotides upstream of the final exon–exon junction on the mRNA, an EJC most likely remains bound at this exon-exon junction, a constellation that is well known to activate NMD [45]. It is thought that in this situation the EJC-bound UPF3B, via UPF2, recruits UPF1 and promotes SURF complex formation and NMD activation.

The observation that ribosomes reside longer at TCs on transcripts subject to NMD [46,47] indicates that NMD-triggering translation termination is mechanistically distinct from proper termination. However, the mechanistic differences between proper and NMD-triggering termination have remained elusive so far.

After formation of the SURF complex on an NMD targeted mRNA and subsequent SMG1-mediated phosphorylation of UPF1, RNA degradation can be induced in several different ways in mammals by the recruitment of the endonuclease SMG6, the SMG5-SMG7 heterodimer, and/or the decapping enhancer proline-rich nuclear receptor co-activator 2 (PNRC2) to phosphorylated UPF1. SMG6 cleaves the mRNA endonucleolytically near the terminating ribosome [32,48,49], while the SMG5/SMG7 heterodimer recruits the CCR4–NOT deadenylase complex, which catalyzes polyA tail shortening and eventually stimulates decapping of the RNA by the decapping complex [30]. A third possibility to induce RNA decay consists in the direct recruitment of the decapping complex by UPF1, either directly or indirectly in a PNRC2-dependent manner [50,51,52,53].

Finally, hydrolysis of the UPF1-bound ATP promotes the release of the NMD factors from the degrading mRNA [54], and concomitantly UPF1 is dephosphorylated by protein phosphatase 2A (PP2A) in a process that seems to require SMG5, SMG6 and SMG7 [55,56].

Thus, in a nutshell, this model proposes that correct translation termination requires the TC to be positioned in the messenger ribonucleoprotein (mRNP) spatially close to termination stimulating factors, such as for example PABPC1, whereas aberrant translation termination would be characterized by the absence of these factors and/or the presence of decay stimulating factors, such as for example UPF1 [2]. From the virus perspective, this general rule can be exploited. As discussed below, viruses have evolved a plethora of strategies to interfere with or utilize NMD to their advantage.

## 5. Viral mRNAs as Substrates for NMD

**Figure 2:**
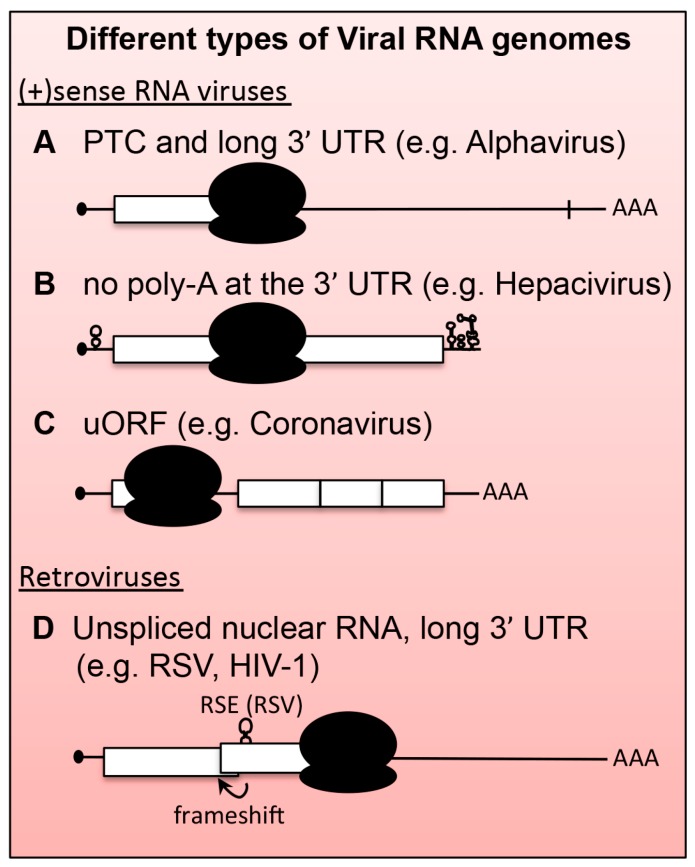
Different types of viral mRNAs that can be substrates for NMD. Similar to aberrant or unconventional cellular mRNA, viral transcripts contain features that make them susceptible to NMD, including the presence of PTC and long 3’-UTRs (**A**); the absence of cap-structure and polyA (**B**); uORFs (**C**); and a combination of all such as in the case of retroviruses RNAs, the translation of which also includes ribosomal frameshifts (**D**). In the case of Rous sarcoma virus (RSV), RNA secondary structures immediately downstream of the TC can act as RNA Stability Elements (RSE; **D**).

During evolution, the replication strategy as well as the selective pressure to maximize the coding capacity of a viral RNA (vRNA) without increasing its genome size has introduced features in vRNAs that could make it a substrate for NMD. In particular, the presence of multiple ORFs on the same RNA makes many of the TCs in vRNAs appear in positions where one would expect them to elicit NMD. Some of the viral ORFs resemble uORFs and many TCs occur in the middle of the transcript far away from a polyA tail. Figure 2 depicts a series of viral mRNAs that contain *bona fide* signatures of NMD recognition (compare Figure 2 with Figure 1). However, vRNAs flourish in the nucleus and cytoplasm of infected cells, suggesting that viruses employ mechanisms that protect their transcripts from the host cell’s degradation pathways.

## 6. Documented Role of NMD and NMD Factors in Virus Infection

To date, a role for NMD or NMD factors in virus infection has been demonstrated for positive‑stranded RNA ((+)RNA) viruses of animals (alphaviruses and hepatitis C virus) and plants (*Alphaflexiviridae* and *Tombusviridae*) [57,58], and for retroviruses (Rous Sarcoma virus, human T‑lymphotropic virus type 1, and human immunodeficiency virus (HIV)) [59,60]. As illustrated in Figure 2 and discussed below, the mRNAs produced by each of these viruses contain features known to trigger NMD. In few cases, a viral counter-defense mechanism has been identified that protects the viral mRNA from NMD.

### 6.1. Positive-Stranded RNA ((+)RNA) Viruses

#### 6.1.1. Alphaviruses and Plant (+)RNA Viruses

Recent studies have shown that NMD might constitute a conserved arm of the intrinsic innate immunity that is able to recognize and degrade vRNAs in extant eukaryotes, including mammalian cells, insects and plants [57,58,61]. Genome-wide small interfering RNA (siRNA) screens carried out in mammalian cells identified UPF1, SMG5 and SMG7 as host factors that restrict the replication of Semliki Forest virus (SFV) and Sindbis virus (SINV), two alphaviruses of the *Togaviridae* family [57]. Follow-up studies in cells infected with replication-incompetent viruses showed that depletion of UPF1 increased the half-life of the SFV genomic RNA (gRNA), indicating that this RNA is a substrate for NMD [57,62].

**Figure 3:**
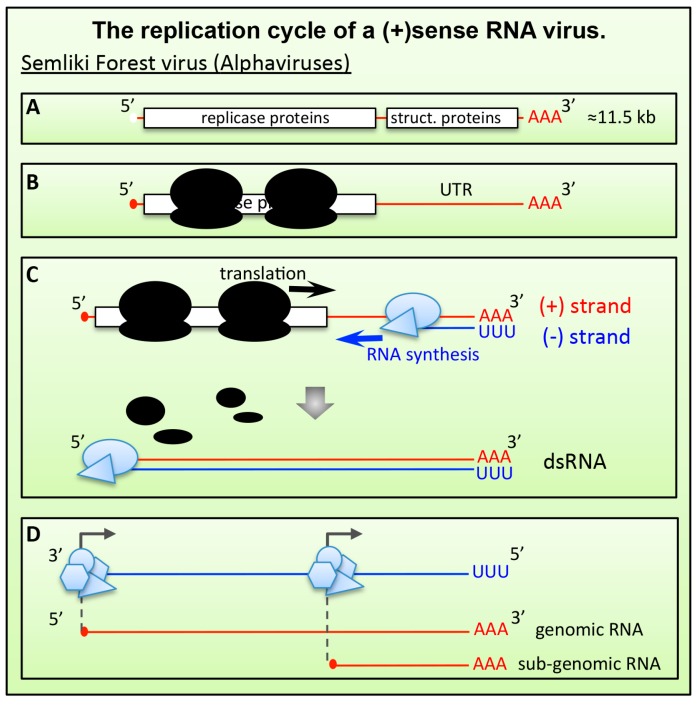
The replication cycle of a positive-stranded RNA ((+)RNA virus). (**A**) The bi-cystronic mRNA genome of Alphaviruses is capped a poly-adenylated. The first ORF encodes the replicase proteins; the second ORF encodes the structural genes (capsid and envelope proteins). (**B**) After virus entry, the genome is delivered into the cytoplasm where the first ORF is translated, leaving a ≈4000 nucleotides long 3’-UTR. (**C**) Newly synthesized viral replicase polyproteins assemble at the 3’-end of the genome and produce a complementary “minus” sense copy. Translation of the viral genome must be shut down. (**D**) After auto-proteolytic cleavage, the replicase complex switches the template and uses the “minus” strand to synthetize new copies of full-length genome and a shorter sub-genomic mRNA that encodes the structural proteins (the second ORF).

As for all (+)RNA viruses, the genome of alphaviruses is an mRNA-like molecule that once released into the cytoplasm of infected cells is immediately translated (Figure 3). The alphavirus genome contains two ORFs. The upstream ORF encodes the nonstructural proteins responsible for genome RNA replication and transcription. The second ORF encodes the structural proteins, capsid and envelope, that are required for the assembly of new virions (Figure 3A). Translation of the second ORF requires the synthesis of a sub-genomic mRNA encoded in the 3′ third of the genome. This configuration creates a very long 3′ UTR (about 4000 nucleotides) during the translation of the first ORF from the full-length gRNA (Figure 3B), which could increase susceptibility to NMD. Surprisingly however, substantial shortening of the 3′ UTR failed to relieve the gRNA from the repressive effect of UPF1. Thus, it is currently not known what renders the genome of alphaviruses susceptible to NMD. In addition to the length of the 3′ UTR, other factors could render the viral mRNA genome a target of NMD. Translation termination is a crucial moment for NMD activation (see Section 4). As for all other (+)RNA viruses, the replication of Alphavirus genomes requires the synthesis of a full-length complementary RNA strand (referred to as the negative-sense RNA ((-)RNA)). The viral replication complexes, encoded by the first ORF, synthesize (-)RNA in 3′‑to‑5′ direction of the gRNA (Figure 3C). Translating ribosomes, however, move along the same RNA molecule in 5′-to-3′ direction, opposite to the viral polymerase complex. This begs the question how the viral polymerase can copy the RNA genome, if the same molecule is used by ribosomes that move in opposite direction? A mechanism must exist to clear translating ribosomes from the vRNA template. The molecular details of this process are not clear. According to current models, the newly synthesized viral replicase proteins could bind to both vRNA and components of the translation machinery to trigger translation termination (or block translation re-initiation). This could create a RNP environment that triggers NMD activation. Consistent with this model, Balistreri et al. showed that impairing the helicase activity of the viral replicase complex rendered SFV hypersensitive to UPF1 depletion, which increased virus replication in primary human cells up to 20-fold [57,63]. More studies are required to shed light on this important aspect of virus replication.

In an independent study, the role of UPF1 as a restriction factor for SINV was confirmed in vivo in insects, using *Drosophila* as a model system [61]. In this study, the two ORFs of SINV were separately inserted into the cellular genome of the insect cells and the transcription of the viral RNAs was launched by an inducible promoter. In this system, inhibition of the RNA interference (RNAi) pathway provided a first means to increase virus production. In addition, expression of a dominant negative form of UPF1 increased the yields of released viruses by more than three-fold. These results are in agreement with those obtained in mammalian cells and collectively indicate that something in the genome of alphaviruses, or in the mode of translation termination, renders these RNA molecules susceptible to UPF1-mediated restriction.

In addition to alphaviruses, antiviral effects of NMD have also been shown for (+)RNA viruses of plants [58]. Starting from a genetic screen in *Arabidopsis*, this study identified UPF1, UPF3 and SMG7 as restriction factors for (+)RNA viruses of the *Alphaflexiviridae* and *Tombusviridae*. Similar to alphaviruses, these viruses also have mRNA genomes with long 3′ UTRs due to the presence of multiple ORFs encoded by subgenomic RNAs. Unlike the results obtained for SFV in animal cells, however, Garcia and colleagues showed that shortening the 3′ UTR of the corresponding viral transcript did increase vRNA accumulation in infected cells and resulted in more efficient virus spread. Thus, for these particular viruses, the length of the gRNA 3′ UTR is an important determinant for their susceptibility to NMD. Supporting this notion, the authors showed that a (+)RNA virus of the *Potiviridae* family with a single ORF and a short 3′ UTR was not restricted by the NMD.

#### 6.1.2. Hepatitis C Virus (HCV)

Recent studies have shown that approximately 3%–10% of the transcriptome is affected by NMD directly or indirectly in diverse eukaryotes [10]. Thus, virus induced global inhibition of the NMD pathway could lead to major rewiring of the cell, a strategy that viruses could use to create an environment favorable for virus infection. Recent evidence suggests that this could be the case for hepatitis C virus (HCV), a leading cause of liver disease. Using hepatoma cell lines, Ramage and co-authors showed that HCV infection causes a progressive inhibition of NMD activity as measured by the accumulation of three cellular RNAs known to be NMD substrates (SC35, ASNS and CARS) [64]. A combination of proteomics and RNAi-screening approaches revealed that the viral protein “core” binds to the EJC recycling factor WIBG/PYM and prevents its interaction with other components of the EJC (Y14 and Magoh). Depletions of WIBG/PYM decreased HCV replication and concomitantly suppressed the accumulation of NMD substrates. The mechanisms by which EJC components and NMD inhibition influence HCV replication remains unclear. Like other (+)RNA viruses that infect mammals, HCV is a cytoplasmic virus. Viral RNAs never reach the nucleus and do not contain introns. The inhibition of NMD observed upon HCV infection could result in the stabilization of multiple cellular mRNAs. This could contribute to creating an environment favorable to virus replication and could contribute to pathological effects associated with HCV infection [64]. Future studies are needed to shed more light into this process.

### 6.2. Retroviruses

#### 6.2.1. Rous Sarcoma Virus (RSV)

Once transcribed from its integrated DNA, the full length unspliced transcript of Rous sarcoma virus (RSV) consists of a single mRNA molecule that contains several ORFs (Figure 2D). Although ribosomes translating the first ORF, *gag*, terminate about 7000 nucleotides upstream of the polyA tail and could therefore be expected to activate NMD, the unspliced full length viral RNA is surprisingly resistant to NMD [65]. However, PTCs experimentally introduced into different places of the *gag* ORF were shown to trigger NMD in chicken cells, suggesting that the natural TC of the *gag* ORF may be protected from activating NMD by specific *cis*-acting signals [65,66]. Indeed, it was found that this NMD resistance is conferred by a 400 nucleotides long sequence element located immediately downstream of *gag* TC that was termed the RNA stability element (RSE, Figure 2D) [67,68]. The RSE forms a complex RNA secondary structure [69] and includes several pyrimidine-rich stretches that were recently shown to serve as the binding platform for the polypyrimidine tract binding protein 1 (PTBP1). Recruitment of PTBP1 to the proximity of a termination codon prevents the recruitment of UPF1 and thereby antagonizes NMD, leading to the stabilization of RSV full length RNA and reporter transcripts [70]. The pyrimidine-rich sites are essential for RNA stabilization as mutations at these sites abolished protections of both viral and reporter mRNAs [70]. The authors of this study further showed that the role of PTBP1 as an NMD antagonizing factor is not limited to viral RNAs but that it can also efficiently protect cellular mRNAs from NMD when recruited downstream of an otherwise NMD-triggering TC.

#### 6.2.2. Human T-lymphotropic Virus Type 1 (HTLV-1)

Stabilization elements such as the RSE of RSV act in *cis* to protect the mRNA molecule that harbors them. As an alternative strategy, two proteins from another retrovirus, human T‑lymphotropic virus type 1 (HTLV-1), have been shown to bind to core components of the NMD machinery, causing a global inhibition of the NMD pathway. In one study, the protein TAX was shown to bind UPF1 and the translation initiation complex component INT6/eIF3E, which resulted in partial inhibition of NMD and the stabilization of viral transcripts [71]. A second study reported that the viral protein REX had similar function and was even more efficient than TAX in inhibiting NMD [72]. In this scenario, the viral proteins act in *trans* to inhibit NMD and in turn increase the half-life of viral mRNAs.

#### 6.2.3. Human Immunodeficiency Virus Type 1 (HIV-1)

In the tug-of-war between pathogens and their host defense mechanisms, viruses often evolve means to subvert the function of cellular factors to their advantage. In the case of human immunodeficiency virus type 1 (HIV-1) for instance, two groups have shown that rather than restricting infection, UPF1 is a positive regulator of virus gene translation, vRNA nuclear export, and specific infectivity of released virions [73,74,75]. One of the groups reported an association and co-localization of UPF1 with the viral structural protein Gag during virus replication [73]. Depletion of UPF1 caused a reduction of the HIV-1 RNA and protein pr55Gag and UPF1 overexpression led to an up-regulation of HIV-1 expression. These UPF1‑mediated effects required ongoing translation and the ATPase activity of UPF1 but not the interaction with UPF2 [73]. Interestingly, overexpression of UPF1 mutants that are inactive in NMD were still found to increase HIV-1 gene expression, suggesting a mechanism that requires the ATPase activity of the cellular enzyme but that is different from the canonical role of UPF1 in NMD. A follow-up study documented the presence of UPF1 in two distinct viral RNPs during the late replication phase [74]. One of them was detected in the cytoplasm and contained UPF1 and the viral protein Gag. The second RNP, containing UPF1, Rev, CRM+, DDX3, the nucleoporin p62, and the short isoform of UPF3a (UPF3aS), was shown to promote nucleo-cytoplasmic export of the vRNA. Interestingly, while the interaction between these RNP components and UPF1 was necessary for nuclear export of the viral genome, UPF2 and the long isoform of UPF3a (UPF3aL) were excluded from this complex. UPF2 was a negative regulator of HIV-1 RNA nuclear export. Unlike UPF3aS, UPF3L binds UPF2 and overexpression of UPF3aL also repressed the nucleus-cytoplasmic transport of the unspliced vRNA [74]. In this elegant work, the authors propose a model in which the viral protein Rev might bind UPF1 and compete for the binding of UPF2, thereby excluding this negative regulator (and its partner UPF3aL) from the viral RNP in the nucleus and promoting nuclear export of the HIV-1 RNA genome. This model implies that the overexpression of UPF2 and similar negative regulators of vRNA nuclear export could be used in conjunction with other approaches as a therapeutic strategy to interfere with HIV-1 replication [74].

Along the same lines, Serquina and colleagues confirmed that UPF1 is a positive regulator of HIV-1 infection, while the NMD-cofactor UPF2 had no role [75]. Following proteomic studies, the authors found that HIV-1 virions contain UPF1 and that viruses produced from UPF1-depleted cells are much less infectious. The loss of infectivity was not due to differences in the structure of released viruses, which contained normal ratios of genomic RNAs and correctly processed structural components. Instead, the authors found that the lack of UPF1 from virus-producing cells resulted in released virions that had impaired reverse-transcription during the following round of infection. Interestingly, the ATPase activity of UPF1 was not necessary for this step. Thus, the positive role of UPF1 on HIV‑1 RNA nuclear export and translation is ATPase dependent [74], whereas the UPF1‑induced increased efficiency of reverse-transcription in newly infected cells is ATPase independent [75].

In cells lacking UPF1 or expressing ATPase-defective UPF1 mutants, the infectivity of HIV-1 virions was impaired [75]. These results indicate a role of UPF1’s helicase activity during virion assembly and release. The results of the two groups differ in that Serquina and colleagues did not detect an increased HIV-1 expression upon exogenous UPF1 overexpression. A possible explanation for the discrepancy between the results is that the two groups monitored different readouts and that the experiments were performed in different cells, which are known to have difference susceptibility to UPF1 depletion.

## 7. Conclusions and Outlook

Thus far, NMD has been viewed as a cellular process that regulates stability of certain types of aberrant mRNAs. Given the recent findings indicating an antiviral role of NMD, it has been speculated that the detection of pathogenic RNAs might have been the selective advantage driving the evolution of NMD rather than quality control of cellular mRNAs [76].

Regardless of its origin, the fact that viral genomes can be detected by NMD and that viral proteins have evolved to counteract/exploit NMD suggest that this pathway plays an important role during virus infection. Viruses will therefore become valuable tools for dissecting how NMD factors interact with and are activated by RNA substrates. Conventional tools, such as plasmid-based reporter constructs and inducible systems, provide RNA molecules that are produced in the nucleus and are therefore influenced by nuclear RNA processing and quality control mechanisms. One of the great advantages of using viruses as a tool to investigate NMD is that for many of them (e.g., (+)RNA viruses), the genome is delivered directly into the cytoplasm of the infected cells without undergoing nuclear quality control. This provides the unique opportunity to study specifically the cytoplasmic events of NMD without separated from any effects on RNA stability originating from splicing, nuclear export and RNA editing. Moreover, identification of viral factors that interfere with NMD and the characterization of the molecular mechanisms of this processes will shed new light onto different steps of the NMD mechanism, such as substrate recognition, UPF1 activation and mRNA degradation.

An important question to be addressed in the future is what triggers NMD in viral RNAs. Although many viral transcripts contain signatures known to activate NMD, a prediction cannot always be made based on the knowledge of known cellular NMD targets. In the case of alphaviruses for instance, the truncation of most of the long 3′ UTR from the viral genome did not relieve the virus from being suppressed by UPF1 [57]. This unexpected result implies that in addition to 3′ UTR length, translation termination of the viral first ORF is aberrant for another reason than the distance to the polyA tail. Future work will hopefully clarify these issues.

In addition to the NMD core components, different viruses have been recently described to specifically inhibit or degrade cellular factors involved in RNA degradation [62], which supports a general antiviral role for the cellular RNA turnover systems and indicates that in the absence of viral counter-defense strategies, viral mRNAs would become detectable and vulnerable to these quality control pathways. Understanding how cells recognize viral transcripts and how viruses avoid being detected by these degradation pathways will be of great help to further our understanding of the NMD mechanism but also possibly contribute to finding new ways of interfering with virus replication and hence prevent diseases.
